# Toward understanding the complexity of long-duration energy storage siting in high renewable power grids

**DOI:** 10.1016/j.isci.2025.112571

**Published:** 2025-05-02

**Authors:** David L. Cole, Sourabh Dalvi, Victor M. Zavala, Omar J. Guerra

**Affiliations:** 1 Chemical and Biological Engineering Department, University of Wisconsin-Madison, Madison, WI 53706, USA; 2 Grid Planning and Analysis Center, National Renewable Energy Laboratory, Golden, CO 80401, USA; 3 Mathematics and Computer Science Division, Argonne National Laboratory, Lemont, IL 60439, USA

**Keywords:** Energy management, Energy modeling

## Abstract

Long-duration energy storage (LDES) devices are not yet widely installed in existing power systems but are expected to play a significant role in high variable-renewable energy grids. Siting LDES devices is complex and can significantly impact system cost, but the factors influencing optimal LDES device placement are not fully understood. We consider the optimal placement of an LDES device in two different power systems with varied system configurations. We analyze the impact of VRE concentration and location, load location, other storage device locations, and transmission availability using production cost models. We find that all of these factors influence optimal LDES siting, and no single metric considered was able to consistently identify optimal placement. Further, we found that LDES placement in the grid can have impacts that propagate throughout the grid (e.g., increasing curtailment in certain areas while decreasing it in others), and we propose several areas of future work.

## Highlights

• System configuration impacts on LDES siting in high VRE power grids is considered
• VRE concentration/location, load location, other storage, and transmission all impact siting
• No simple metric tested (e.g., co-locating with VRE) consistently sites LDES optimally
• Additional models and methods are necessary to optimally site LDES in high renewable grids

## Graphical abstract

**Figure:**
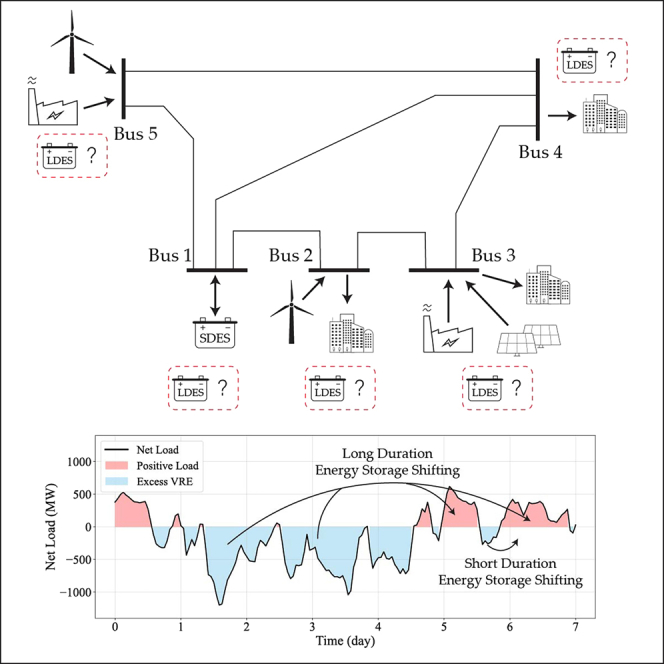

## Introduction

Storage technologies are essential components of high variable renewable energy (VRE) grids as they allow for shifting variable renewable generation in time.^1^^,^^2^ Storage systems can take varying forms^3^^,^^4^^,^^5^ and have varying durations (i.e., the time needed to fully discharge the storage device), including short-duration energy storage (SDES; <10 h of duration), long duration energy storage (LDES; ∼ 10-100 h of duration), and seasonal energy storage (>100 h of duration). Each of these devices can have different roles in the grid (e.g., intra-day vs. inter-day energy shifting)^6^ and play important roles in decarbonizing power systems^7^^,^^8^^,^^9^ and improving system resilience.^10^ However, few long-duration energy storage (LDES) systems have been integrated into existing grids,^11^ and siting of these systems is not well understood. The placement of the same storage device at different locations could alter system production costs by millions of dollars (as we show in this work) and is thus an important factor to consider.

Storage siting decisions play an important role in grid performance and operation, but identifying optimal storage placement is difficult.^1^^,^^12^ Siting storage can require balancing competing objectives,^12^ including minimizing system operator cost, maximizing storage owner profit,^13^^,^^14^^,^^15^^,^^16^^,^^17^^,^^18^ or reducing transmission requirements.^19^^,^^20^^,^^21^^,^^22^ For example, deploying storage on congested transmission corridors could provide a means for providing virtual transmission capacity^23^ and/or deferral of transmission upgrades. Building new transmission resources can take a decade or longer^24^^,^^25^ and can face strong local opposition,^26^ so storage can provide a near-term solution to expanding transmission assets. However, siting storage with the sole goal of reducing transmission congestion may not align with the location that best decreases overall system cost. Also, accurately simulating large scale power systems containing storage can be computationally expensive, and it is often not possible to model all possible siting decisions and scenarios. While some have proposed general siting heuristics (e.g., siting storage near high-energy demand or generation facilities, especially VRE^27^), it is possible that simple metrics do not capture the true complexity of the problem at hand. Therefore, understanding what factors can contribute to or be impacted by storage siting is valuable in balancing competing interests and/or informing stakeholders.

Several studies have considered siting of energy storage devices. For example, Wong et al.^12^ reviewed over 15 different studies that considered siting energy storage based on analytical and optimization-based methods. Of these, the largest case study appears to be from Fernández-Blanco et al.^1^ who introduced a mixed integer linear programming (MILP) approach for both sizing and siting storage systems in a 240-bus grid. Other studies have also been done since their review, including Boonluk et al.,^28^ Gupta et al.,^29^ and Peña et al.,^30^ which likewise used MILP formulations for sizing and siting. However, to our knowledge, the existing literature in this area does not focus specifically on LDES devices, and no existing study considers a wide variety of system configurations to test impacts of system components on siting decisions (e.g., how does the location of a wind generator impact siting decisions), limiting the studies’ ability to be applied to other systems.

In this work, we consider impacts of grid configuration on optimal LDES siting and how siting decisions impact system operation. We do this by identifying optimal LDES locations for many different system designs (e.g., location of VRE, loads, or other storage devices) and observing the impacts of configuration on optimal LDES locations. We focus primarily on LDES, but the results are connected to SDES components and could potentially extrapolate to seasonal storage. We also identify important areas of future work for these topics. Overall, VRE location and concentration, load location, other storage, transmission availability, and network topology all appear to play a role in optimal siting of LDES systems. Our results indicate that simple metrics for LDES siting (e.g., co-locating with VRE) can result in sub-optimal siting decisions, and further work needs to be done to better correlate system configurations and optimal siting locations. In addition, we observe that additional computational tools could be developed for addressing challenges like storage placement in a more efficient manner, such as developing new tools for working at the interface of capacity expansion and production cost models.

## Results and discussion

### 5-Bus system

**Figure 1:**
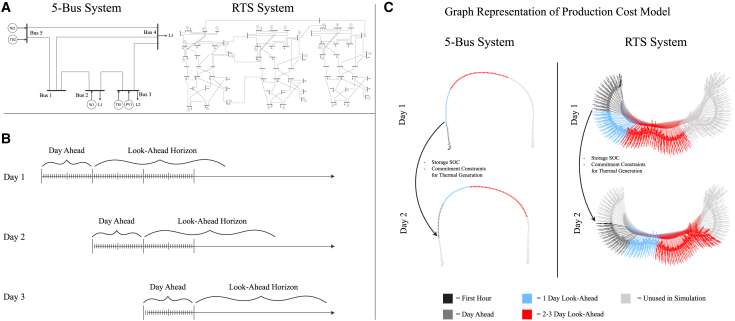
Production Cost Models Visualization Visualization of (A) the 5-bus system and RTS-GMLC used in the production cost models. The 5-bus system shown is an adapted visual from Li et al.^42^ and the RTS-GMLC shown is adapted from González-Fernández et al.^43^ (B) shows how PCMs operate with a day-ahead span (this is the solution used in practice) with some look-ahead horizon that is moved forward after each simulation. (C) Shows an alternative visualization of the PCM to highlight complexity. The PCM solved in Sienna^44^ can be represented as a graph that captures both the spatial and temporal representation of the optimization problem. Here, nodes represent buses and transmission lines in the system with edges placed between nodes when a constraint of the PCM considers both nodes. A simulation of the PCM consisted of 350 days solved in sequence. Each simulation used a 1-h time resolution and solved for the day-ahead operation (1 day). The simulation also included some look-ahead horizon (shown by the blue and red highlighted nodes and edges) which helps inform the decisions of the day-ahead period. After a single day (1 day-ahead) was solved, the simulation took a step forward in time and solved the next day-ahead period using the previous solution’s data for the storage state of charge (SOC) and thermal generators’ statuses. Simulations without LDES used 1 day of look-ahead while simulations containing LDES used 14 days and 3 days of look-ahead for the 5-bus system and RTS-GMLC, respectively. See Figure S1.

**Figure 2:**
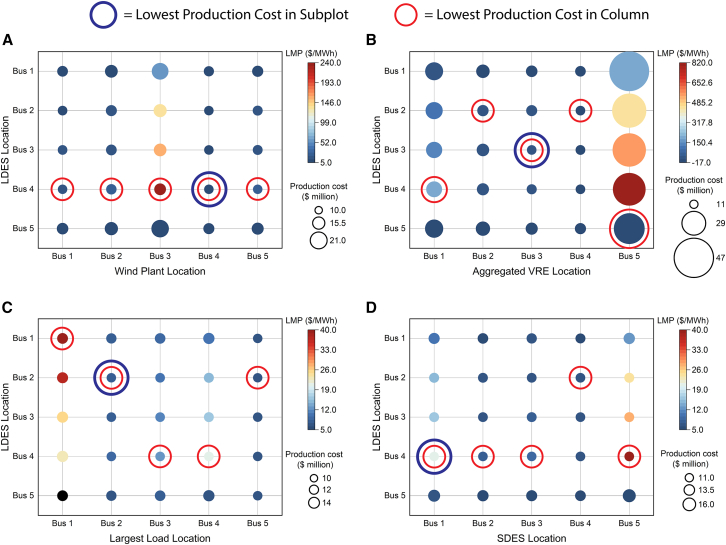
5-bus system simulation results Results of PCM simulations in Sienna for the 5-bus system. The horizontal axis shows what factor was changed and where system components were placed in the simulation, and the vertical axis shows the location of LDES for a given simulation Bubble sizes show production cost (with all bubbles to scale across the plots), bubble shading shows average locational marginal price (from the simulation with no LDES, as this information could be used for identifying optimal deployment locations), the blue circled node in each subplot represents the overall lowest production cost, and the red circled node in each column represents the simulation with the lowest production cost for that set of simulations (i.e., the optimal siting location based on production cost). System configurations that were changed include (A) moving the wind plant from Bus 5 to other buses in the system; (B) placing all three VRE generators in the system on a single bus; (C) moving the largest load (originally on Bus 4) to a different bus in the system; (D) moving the SDES to a different bus (originally on Bus 1). Standard deviations for LMPs are reported in Table S2.

The 5-bus and reliability test systems (RTS-GMLC) are visualized in Figure 1. The results of the 5-bus system analysis are shown in Figure 2 and suggest that VRE, load, and SDES location all had some influence on optimal siting of the LDES.

When VRE was dispersed (Figure 2A), the location of individual VRE plants did not change the optimal siting of the LDES (Bus 4). For systems with VRE dispersed throughout or with few transmission constraints, VRE location alone may not be the best indicator for location of LDES. In contrast, when VRE is concentrated in the same bus (Figure 2B), its location corresponded with the optimal LDES location in 3 of the 5 configurations. This shift from the dispersed case (Figure 2A) may be due to transmission constraints in the system which are exacerbated by the more centralized VRE. In the aggregated case, there are hours where the combined VRE production is greater than any bus has capacity to export. Buses 2, 3, and 5 have less interconnection capacity than buses 1 and 4, so LDES likely plays a greater role in decreasing curtailment when it is co-sighted with the aggregated VRE on this bus. The level of concentration of VRE can impact optimal LDES siting, and co-locating with VRE may be best when transmission capacity is limited. Similarly, co-locating with largest load location also did not always correspond to optimal citing (Figure 2C). This may be due to sufficient interconnection capacity and the ability for certain buses to move power to other buses. For SDES siting impacts (Figure 2D), if SDES is sited at the same time as LDES (i.e., the lowest production cost in the 5x5 grid), the optimal siting is SDES on Bus 1 and LDES on Bus 4. However, for many systems, SDES may already be constructed in the system, and this could impact LDES siting. For instance, if SDES was already deployed on Bus 4, the optimal LDES siting would be Bus 2. Therefore, SDES and LDES may best be sited simultaneously to leverage synergistic effects.

In 13 of the 20 columns of Figure 2, the largest average locational marginal price (LMP; average at a bus over all simulated days) also corresponded to the optimal location of LDES, and LMPs could be used for identifying LDES placement (note that the largest standard deviation of LMP always corresponded to the largest average LMP). Thus, average LMP may correlate to optimal siting locations, but it alone is not enough to identify optimal LDES placement. This could be in part because the average LMP may be driven by a small percentage of times where the LMP is many times larger than usual and may raise the average disproportionately. Further, the largest average LMP was almost always on Bus 4, meaning the location of the largest average LMP rarely changed with system configuration (see Table S1).

The VRE mix could also play an important role in optimal storage siting and would be an important factor to consider in the future. For instance, in Figure 2A, swapping the wind generator at Bus 5 with the PV generator at Bus 3 resulted in a 20% increase in production costs versus the original configuration with LDES sited optimally in both systems. This is likely because Bus 5 also contains the cheapest thermal power available in the system and experiences more transmission congestion during peak hours with solar than with wind co-located with the thermal generator.

We also considered the impact of transmission capacity on LDES siting. We reduced the system’s transmission capacity (each line was reduced by the same percentage) and ran simulations with LDES sited on a different bus. Transmission capacity reductions of 10%, 20%, and 30% did not change the optimal siting location (Bus 4), but a 40% reduction resulted in Bus 3 becoming the optimal location for the LDES, with a cost more than 5% lower than placing it on Bus 4 (see Table S1). This may be because Bus 3 is more centrally located for the loads (on buses 2, 3, and 4) while also being located near or with much of the VRE (on buses 2 and 3). Bus 4 may have greater difficulty in supplying power to meet the load on Bus 2 once the transmission capacity is reduced by 40%. Further, this could mean optimal siting of storage may need to be informed by expected future developments, as future deployment of VRE or future construction of transmission could influence congestion at or near the storage device. Optimal siting of storage today may not be optimal siting of storage 5 years from now.

### RTS-GMLC

**Figure 3:**
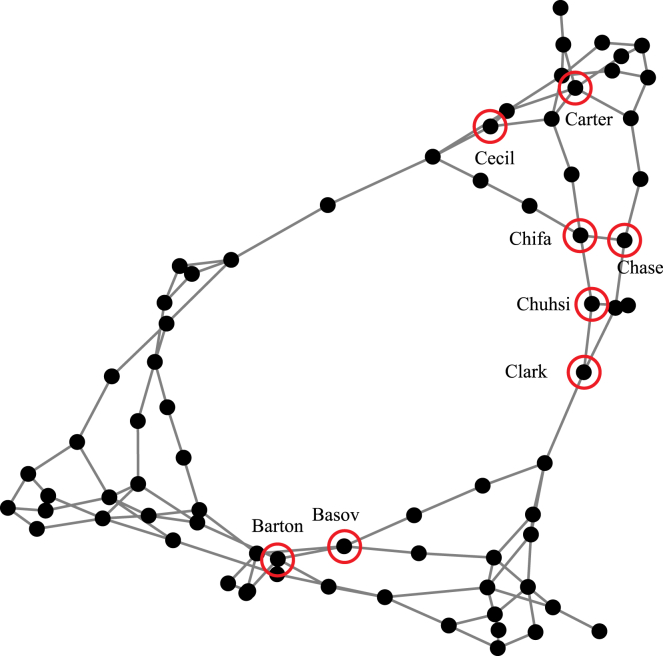
RTS-GMLC Visualization Visualization of the RTS-GMLC where nodes represent buses and edges represent transmission lines or tap transformers. Eight different locations were tested for LDES siting (Table 1), and each of these locations is labeled and highlighted by a red circle. Node locations are determined by graph topology and do not represent spatial scales.

For validating the results of the 5-bus system, we also ran simulations on the larger, RTS-GMLC. The results of these tests are presented in Table 1, with Figure 3 showing the bus locations for the RTS-GMLC, where each node in the figure represents a bus and edges between buses represent transmission lines or tap transformers. The RTS-GMLC provides a more practical example of the various challenges observed when it comes to optimally siting of LDES. Co-locating with the largest VRE (Bus Chuhsi) resulted in the worst performance (based on production cost) of our simulations containing LDES, followed by locating at the bus with the largest closeness centrality (Basov), followed by co-locating with the largest net load (Chase) and load (Clark). Of the five singular metrics we tested, siting LDES at the bus with the largest average LMP (Carter) was best. However, the two best overall solutions of our simulations by a large margin ($25+ million or 12+% less production cost than the next best options) were placing LDES on a bus containing high VRE and high transfer capacity (Bus Chifa) and/or little congestion (buses Chifa and Cecil). Despite having a larger transfer capacity, Bus Barton was a worse siting location than buses Chifa or Cecil. Nearby buses may play a role in optimal placement as Bus Chifa is connected directly to Bus Chase (with the largest net load), while Bus Barton is placed in the same region as an SDES device. In addition, buses Chifa, Cecil, and Barton only contain solar PV generators while Bus Chuhsi only contains wind generators, and the VRE mix at each node could be an important factor in optimal LDES siting. Overall, we found that no single tested metric provided an optimal siting decision.

**Table 1:** RTS-GMLC results

Bus Name	Bus Characteristic	Transfer Capacity (MW)	# Congested Hours at Bus	Production Cost (Million USD)	Cost Percent Reduction
–	No LDES	–	–	288.4	–
Clark	Largest Load	2,000	656	228.1	26%
Chase	Largest Net Load	2,000	1	230.5	25%
Chuhsi	Largest VRE	1,500	587	244.4	18%
Carter	Largest Avg. and Std. Dev. LMP	1,325	264	222.7	30%
Basov	Largest Closeness Centrality	2,000	0	231.1	25%
Chifa	5^th^ highest VRE, next to Largest Load	2,000	99	195.9	47%
Cecil	4^th^ highest VRE, no congested hours	1,500	0	196.4	47%
Barton	13^th^ highest VRE, 3^rd^ highest transfer capacity	2,500	0	233.3	24%

Results from the RTS-GMLC simulation, where the LDES is sited at a different bus. The “Bus Characteristic” indicates the reason for siting the LDES at this location, either as data from the RTS-GMLC or from the simulation of the RTS-GMLC without any LDES. The “Transfer Capacity” is the transmission capacity of all connected transmission resources. The “# Congested Hours” is the number of hours during the simulation (with no LDES) that a connected transmission resource was at 100% of capacity (normalized by the number of connected resources) at the given bus. Cost Percent Reduction is compared to the no LDES case.

Further work needs to be conducted to address the challenge of siting LDES and identify metrics for optimal LDES siting. No single metric we tested could indicate ideal siting locations, and a more accurate metric is likely a combination of the factors discussed above as well as network topology and potentially other factors (such as amount of time a bus sees congestion). In addition, we used a limited look-ahead horizon with LDES (3-day), and the LDES operation may not have been fully optimal because it could not “see” far enough into the future, but using larger look-ahead horizons is not computationally feasible.^31^ However, it was observed in Guerra et al.^31^ that using 3-day rather than 1-day look-ahead does better capture the value of the LDES. Unfortunately, it is also not practical to simulate LDES placement on all 73 buses in the RTS-GMLC using production cost models (PCMs; note that RTS-GMLC simulations took 1–2 days each to run), meaning it would be important to develop other models that could help answer the question of optimal placement or identify better metrics for indicating optimal placement.

**Figure 4:**
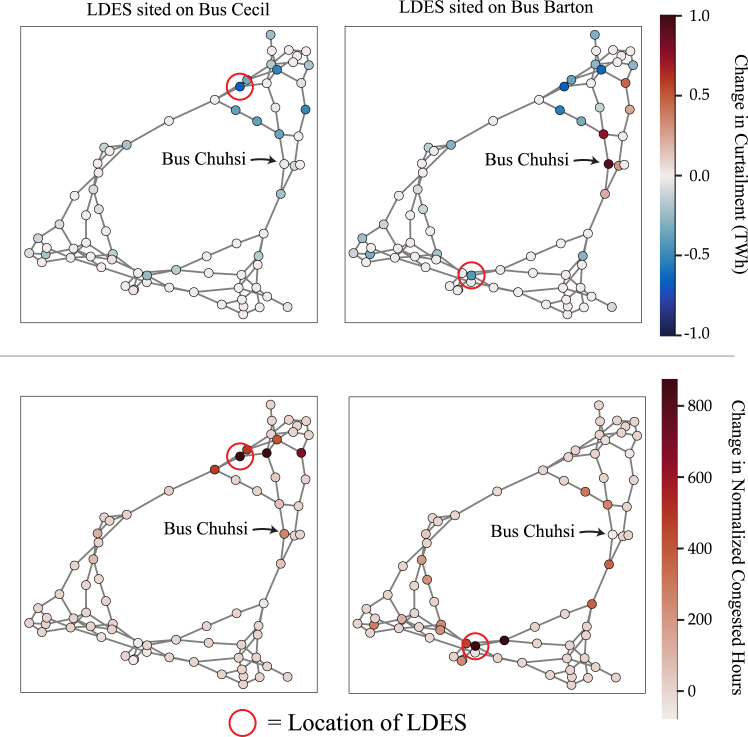
RTS-GMLC LDES Siting Comparison Change in curtailment (top) and in normalized congested hours (bottom) by bus. For the top, red represents more curtailment at that node and blue represents less curtailment at that node as compared to the case with no LDES. For the bottom, darker shading represents an overall increase in normalized curtailment as compared to the case with no LDES.

Lastly, it is important to evaluate and better understand how the impacts of LDES propagate throughout the grid. While both buses Cecil and Barton resulted in decreasing curtailment in the system compared to the case with no LDES, different buses in the system saw different impacts to their curtailment and congestion based on LDES siting (see Figure 4) For instance, siting LDES on Bus Cecil resulted in overall curtailment decreasing by 49%; however, the VRE generators on Bus Chuhsi saw combined curtailment decrease by only 1.7% (Figure 4). In contrast, when LDES is sited on Bus Barton, overall curtailment decreased by 26% but VRE generators on Bus Chuhsi saw curtailment *increase* by 59%. Further, transmission operation changed with the addition of LDES that was not necessarily consistent throughout the grid—placing LDES at Bus Barton increased the number of normalized congested hours at Bus Chifa from 99 h to 359 h while at Bus Chuhsi, they *decreased* from 587 h to 509 h, and this despite Bus Chifa being farther from Bus Barton than Bus Chuhsi (see STAR Methods for details on “normalized congested hours”). In contrast, locating LDES at Bus Cecil saw congested hours at Bus Chuhsi *increase*, rather than decrease (Figure 4). In other words, the impacts of LDES siting can propagate throughout the grid and could have impacts that are non-intuitive (i.e., one might expect adding LDES would decrease curtailment at every bus, but it actually could *increase* curtailment at some areas).

As it was not computationally feasible to run the RTS-GMLC with a 2-week look-ahead, it is important to validate using a 3-day look-ahead window. To address this, we considered the impacts of look-ahead horizon on the 5-bus system and ran five different simulations with 1-day, 3-day, and 2-week look-ahead horizons and compared the results. Each simulation sited LDES on a different bus (SDES was always sited on Bus 1). The resulting production cost for each simulation is given in Table 2. The production costs from 1-day to 3-day look-aheads all improved by a minimum 6.9% (LDES sited on Bus 5), and often by over 10%. Using a 2-week look-ahead further improved every simulation, but less significantly than did going from 1-day to 3-day look-ahead horizons. What is more, the trends in prices stayed the same regardless of which look-ahead horizon was used—Bus 5 was consistently the worst siting location while Bus 4 was consistently the best. In addition, we ran additional simulations of the RTS-GMLC with LDES sited at Bus Cecil. Using 1-day, 3-day, and 4-day look-aheads resulted in production costs of 200.1, 196.4, and 195.7 million USD, respectively. The added two days of look-aheads (from 1 to 3 days) resulted in a 1.8% improvement, while adding one additional day (from 3 to 4 days) only had a 0.3% additional improvement. This suggests that, while not perfect, the 3-day look-ahead horizons better capture LDES operation and value compared to the 1-day look-ahead horizon and that, while longer look-ahead horizons result in better performance, the 3-day look-ahead is capturing a significant part of LDES device value.

**Table 2:** 5-Bus look-ahead comparisons

LDES Location	1-Day Look-ahead	3-Day Look-ahead		2-Week Look-ahead	
	Production Cost (Million USD)	Production Cost (Million USD)	Decrease (1-Day LA)	Production Cost (Million USD)	Decrease (1-Day LA)
Bus 1	14.54	13.25	8.9%	13.13	9.7%
Bus 2	13.47	12.05	10.5%	11.84	12.1%
Bus 3	13.34	11.89	11.1%	11.66	12.8%
Bus 4	12.98	11.62	10.5%	11.27	13.2%
Bus 5	15.24	14.19	6.9%	14.13	7.3%
Average	13.92	12.60	9.5%	12.41	10.9%

Comparison of look-ahead horizons (1-day, 3-day, and 2-week) for the 5-bus system with LDES sited on a different bus each time. Production costs are reported in million USD and the percent decrease from the 1-day look-ahead horizon is reported for the 3-day and 2-week look-ahead horizon results.

The results of on the RTS-GMLC also highlight the need for effective models for informing these siting decisions. The PCMs run above were already computationally expensive, and we were unable to perform simulations for siting LDES at each bus in the system. It could be beneficial to develop models that can better perform simulations like those above, but perhaps making simplifications (e.g., using a coarser time horizon) while still capturing network topology (e.g., incorporating power transfer factors as we did in this study). The siting of LDES lies at the interface of PCMs and capacity expansion models, and it could be beneficial to develop modeling tools that operate at that interface.

### Future work

The results presented herein suggest several areas of future work. First, more work can be done to identify rules for optimal siting of LDES in larger systems. Ideally, metrics for a bus (e.g., a function of net load, transmission capacity, storage sizing, LMP, etc.) could be identified that correlate to a bus’s candidacy for siting LDES or even seasonal storage. The simple metrics tested in this work failed to consistently identify optimal placement, likely because there are several factors that can impact the optimal siting location. LMPs correlated better than net load or VRE but still left room for improvement. Future work could explore a metric that is a combination of mean or standard deviation of LMP, net load, VRE integration, and topological information, where topological descriptors could be derived from a combination of network structure and data stored on the network (e.g., congestion on edges).^32^ These metrics could also be influenced by storage sizing, and further work could be done to better understand storage sizing with siting^33^ as varying sizes of storage systems will be required in future grids.^6^ For instance, LDES devices can have longer durations than what was tested above, and these systems (or even seasonal storage devices) need further work done to fully understand the impacts of their siting; however, some of these devices, like seasonal storage devices, require more expensive simulation of operation which can span multiple years or weather conditions. The impact of storage on transmission is also important for understanding how storage (SDES, LDES, and seasonal) would impact transmission operation or be used to reduce congestion in high renewable power grids. Another key consideration is how the addition of storage to a system generates effects that propagate throughout the system, and how to determine which locations will be most impacted by the addition of large storage systems. Such impacts are important for understanding system resilience, such as how the system would be influenced by extreme events. For example, in an outage, how “close” does LDES need to be to the outage to help mitigate the outage’s effects?

In addition, there are several modeling questions that would be important to address in the future. PCMs (as used in this work) typically optimize from the grid operator’s standpoint, and the optimal siting for the grid operator may not correspond to the optimal siting for the storage owner as the storage owner would operate in a way to maximize their profit rather than minimize grid operating costs. Further, optimal storage siting (especially LDES and seasonal) would be difficult to compute in larger scale systems as it is not feasible (based on todays’ computational capacities) to simulate large scale systems with LDES sited on every bus. Advances in computing hardware (such as solvers for GPUs^34^^,^^35^), improved solution or decomposition schemes, or development of new modeling approaches could help address this bottleneck. For instance, the question of siting storage lies at the interface between PCMs and capacity expansion models, and it would be valuable to create models that operate at this interface for larger systems. For instance, such models could use coarser time resolutions or different network representations, and identifying the required time and spatial resolution or system constraints to capture the value of storage siting and sizing would be valuable to pursue in future studies.

### Limitations of the study

Some limitations exist in this study scope. First, the 5-bus system and RTS-GMLC were smaller than real operating grids today, and additional work could be done to extrapolate and validate these results for larger systems. For reference on system size, the peak loads seen in these systems were roughly 1–10% of those of larger independent system operators in the United States in 2019.^36^ Smaller systems were necessary for our analysis as we ran more than 100 simulations of the 5-bus system and 9 simulations of the RTS-GMLC to compare the role of system configuration on optimal LDES siting, and this number of simulations would not have been possible in the same amount of time with larger systems. Second, we did not consider different VRE mixes within the same system (e.g., solar-driven vs. wind-driven systems) which could influence operation of storage. Third, we did not consider multiple LDES devices being sited in the grid, and the question of siting multiple LDES devices could lead to additional complexity. Finally, the LDES device tested in this study was on the smaller end of LDES devices, and further work would be required to understand how these results extrapolate to larger devices. Seasonal storage was not directly considered in this study and may require other computational models for accurately capturing its operation and determining optimal placement of these larger devices.

There were also some limitations in the modeling of this study. PCMs focus on minimizing cost to the system operator, not maximizing device owner profit, which could result in different optimal siting decisions from the different entities’ perspectives. It could be further beneficial to consider the markets in which LDES is operating. In this work, we focused on energy arbitrage. Various works focus on energy arbitrage or suggest that the market for arbitrage can be a profitable area for LDES,^16^^,^^17^^,^^18^^,^^37^^,^^38^ but LDES could play some role in ancillary power markets as well.^39^^,^^40^^,^^41^ We also do not consider sizing of batteries or cycling and capital costs associated with the batteries which adds an additional layer of complexity to the modeling.

Lastly, for the RTS-GMLC, we were limited to a 3-day look-ahead horizon for simulations, which could result in suboptimal behavior of the LDES operation. As shown in Table 2, a shorter look-ahead horizon (1-day or 3-day) for the 5-bus system resulted in similar results in terms of optimal siting locations as using a 2-week look-ahead horizon, which suggests that the results of the 3-day look-ahead horizon for the RTS-GMLC gives meaningful (though likely imperfect) results. However, the 5-bus system is a smaller system, and being unable to run the RTS-GMLC with longer look-ahead horizons is a limitation.

## Resource availability

### Lead contact

Requests for further information and resources should be directed to and will be fulfilled by the lead contact, Omar J. Guerra (omarjose.guerrafernandez@nrel.gov).

### Materials availability

This study did not generate new unique materials.

### Data and code availability

• All data used in this study is publicly available and can be accessed at https://doi.org/10.11578/dc.20250402.1. Data includes the 5-bus system and RTS-GMLC systems.
• All code used in this study is publicly available and can be accessed at https://doi.org/10.11578/dc.20250402.1. Code includes scripts for running and analyzing the simulations described in this study.
• Any additional information required to analyze the data reported in this paper is available from the lead contact upon request.

This paper uses publicly available data and code that can be accessed at the links provided in the STAR Methods and key resources table.

## Acknowledgments

This material is based upon work supported by the U.S. Department of Energy, Office of Science, under contract number DE-AC02-06CH11357 and by the U.S. National Science Foundation under award CBET-1748516. We would also like to thank Drs. Bri-Mathias Hodge and Wesley Cole for their helpful feedback.

## Author contributions

D.L.C.: Methodology, software, formal analysis, writing – original draft, visualization; S.D.: Methodology, software, writing – review and editing; V.M.Z.: Funding acquisition, writing – review and editing; O.J.G.: Conceptualization, supervision, writing – review and editing.

## Declaration of interests

The authors declare no competing interests.

## STAR★Methods

### Key resources table

**Table:** 

REAGENT or RESOURCE	SOURCE	IDENTIFIER
**Deposited data**		

5-bus system and RTS-GMLC data	NREL	https://doi.org/10.11578/dc.20250402.1

**Software and algorithms**		

Julia	JuliaLang.org	https://julialang.org/
Xpress Solver	FICO	https://www.fico.com/en/products/fico-xpress-optimization
Gurobi Solver	Gurobi Optimization, LLC	Gurobi.com
HiGHS Solver	HiGHS Linear Optimization Software	Highs.dev
PowerSystems.jl	NREL	https://doi.org/10.5281/zenodo.10835983
PowerSimulations.jl	NREL	https://doi.org/10.11578/dc.20240311.3
InfrastructureSystems.jl	NREL	https://doi.org/10.11578/dc.20240425.2
Code	NREL	https://doi.org/10.11578/dc.20250402.1

### Method details

We designed experiments to analyze i) the impacts of system configuration on LDES and SDES siting and ii) the impacts of storage siting on grid operation and cost. These experiments were performed by running a production cost model (PCM) in Sienna,^44^ which is a suite of Julia packages for general modeling and simulation of power systems. PCMs simulate grid operation and provide the cost of operating the system over the simulated period. Unlike capacity expansion models, PCMs often better capture operational constraints (e.g., network constraints and congestion) because capacity expansion models must operate over a longer time horizon and simplify some constraints as a computational necessity. Sienna provides flexibility in changing system structure and components, allowing us to alter aspects of the system and estimate their impact on the production cost, including simulating operation with and without LDES to identify the cost reduction from LDES being integrated into the system. All simulations ran over a 350-day time span (a full year could not be used because two additional weeks of look-ahead were required for some simulations) and operated under network constraints (DC approximations) for power flow between buses. Sienna readily supports moving the same component (e.g., the LDES device) to different buses in different simulations.

In Sienna, the costs are primarily driven by thermal generator’s operating cost, start-up costs, and shut-down costs, with penalties for under-serving or over-delivering load. Sienna provides significant user flexibility in constructing the PCM, including setting network models. For our simulations, we used the Power Transfer Distribution Factors (PTDF) Network model implemented in Sienna, which is a scalable DC approximation of transmission constraints, allowing us to solve each simulation using only a MILP solver.

PCMs typically simulate a specific time interval, but this can be dependent on the system of interest. Traditionally, PCMs solve a unit-commitment and economic dispatch problem at a 1-h resolution for a single day operation (the day-ahead period) and then have an additional day included as a look-ahead horizon which allows the simulation to see expected future needs as well. After a given day’s simulation completes, the simulation moves forward in time by one day and the process is repeated where the solution of the previous day-ahead period is used as the initial conditions of the next day simulations (e.g., generator commitment decisions of the previous day are used in the up-/down-time constraints of the next day’s simulations and the final energy value of a device of the previous day is enforced as the initial energy value of the device in the next day simulation). When solving PCMs with no storage systems or only short-duration energy storage (SDES) systems, we used only a one-day look-ahead window for each simulated day.

Scripts for performing the numerical experiments below are available at Cole et al.^45^ These include scripts for changing the system configurations in the 5-bus system case and for running the RTS-GMLC simulations are all available as well as scripts for querying results from simulations. Simulations can be performed using different solvers, but our results used HiGHS, Gurobi, and Xpress, depending on the simulation. Different solvers were used depending on the availability of licenses at the times of the simulations.

The two systems used are described below and in tables below. Note that there is both a PV-driven and a wind-driven form of both systems, though we used the wind-driven 5-bus system and PV-driven RTS-GMLC for this study. The systems are also visualized in Figure 1 along with an example of how PCMs use a look-ahead horizon in the optimization horizon. Note that, especially for LDES, the choice of look-ahead horizon length is a significant decision in simulating power system operation.^31^^,^^46^

#### System power overviews

Descriptions of the 5-bus system and RTS-GMLC’s load and variable renewable energy (VRE) as well as installed VRE capacity. Both systems have a PV-driven configuration and a wind-driven configuration. Values reported are for 366 days of data.

**Table:** 

	Total Load (GWh)	Total VRE (GWh)	Total PV (GWh)	Total Wind (GWh)	VRE Capacity (GW)	PV Capacity (GW)	Wind Capacity (GW)
5-bus (PV)	5,780	4,180	2,460	1,720	1.57	1.12	0.45
5-bus (Wind)	5,780	6,040	1,640	4,400	1.90	0.75	1.15
RTS (PV)	37,650	32,980	23,340	9,640	12.85	9.54	3.31
RTS (Wind)	37,650	42,880	11,400	31,480	15.95	4.61	11.33

#### System component overviews

Descriptions of the 5-bus system and RTS-GMLC’s components including loads (# Loads), number of PV generators (# PV), number of wind generators (# Wind), number of thermal generators (# Thermal), number of transmission lines (# Lines), and number of tap transformers (# Tap Tr.). Both systems have a PV-driven configuration and a wind-driven configuration.

**Table:** 

	# Loads	# PV	# Wind	# Thermal	# Lines	# Tap Tr.
5-bus (PV)	3	3	1	2	6	0
5-bus (Wind)	3	1	2	2	6	0
RTS (PV)	51	58	5	54	105	15
RTS (Wind)	51	29	18	54	105	15

#### System SDES overviews

Descriptions of the short duration energy storage (SDES) device contained in the 5-bus system and RTS-GMLC. Both systems have a PV-driven configuration and a wind-driven configuration, and all systems and configurations have only one SDES device.

**Table:** 

SDES	Size (MWh)	Max Charge (MW)	Max Discharge (MW)	Charge Efficiency	Discharge Efficiency	Duration (hr)
5-bus (PV)	576	160	160	0.85	1.0	3.6
5-bus (Wind)	900	250	250	0.85	1.0	3.6
RTS (PV)	3,600	1,000	1,000	0.85	1.0	3.6
RTS (Wind)	5,400	1,500	1,500	0.85	1.0	3.6

#### System LDES overviews

Descriptions of the LDES device contained in the 5-bus system and RTS-GMLC. Both systems have a PV-driven configuration and a wind-driven configuration, and all systems and configurations have only one LDES device.

**Table:** 

LDES	Size (MWh)	Max Charge (MW)	Max Discharge (MW)	Charge Efficiency	Discharge Efficiency	Duration (hr)
5-bus (PV)	5,130	380	380	0.7	1.0	13.5
5-bus (Wind)	8,100	600	600	0.7	1.0	13.5
RTS (PV)	54,000	4,000	4,000	0.7	1.0	13.5
RTS (Wind)	44,550	3,300	3,300	0.7	1.0	13.5

*5-Bus System* (adapted from Li et al.^42^) – This system contains 366 days of hourly data with wind generators on Buses 2 and 5 (combined 1.15 GW of installed capacity, 4,400 GWh of annual generation), a solar generator on Bus 3 (0.75 GW of installed capacity, 1,640 GWh of annual generation), and two thermal generators (520 MW at Bus 3 and 600 MW at Bus 5). Loads were located on Buses 2, 3, and 4 (5,780 GWh of combined annual load; peak demand of 1.5 GW). The LDES device had a 600 MW power capacity with 13.5 h of duration (duration refers to the amount of time a storage system can continuously provide its max power capacity to the grid) and a 70% charging efficiency and the SDES device had a 250 MW power capacity with 3.6 h of duration and an 85% charging efficiency. Note that Sienna is a general modeling framework and the LDES device is not meant to represent a specific technology and is constrained by device parameters like maximum energy levels, efficiencies, and maximum charging or discharging values. This system is much smaller than real operational power grids, but the size allowed us to run dozens of system configurations. Simulations of the 5-bus system with LDES used a 2-week look-ahead horizon; all other simulations used a 1-day look-ahead horizon.

*Reliability Test System (RTS-GMLC)* (adapted from Barrows et al. and González-Fernández et al.^43^^,^^47^) – This system contained 366 days of hourly data with 73 buses, 5 wind generators (combined 3.31 GW of installed capacity, 9,640 GWh of annual generation), 58 PV generators (combined 9.54 GW of installed capacity, 23,340 GWh of annual generation), 54 thermal generators (9.1 GW of total generation capacity), and 51 loads (37,650 GWh of combined annual load; peak demand of 8.2 GW). The LDES device had a 4 GW power capacity with 13.5 h of duration and a 70% charging efficiency and the SDES device had a 1 GW power capacity with 3.6 h of duration and a 85% charging efficiency. Simulations with LDES used a 3-day look-ahead horizon (larger look-ahead horizons were too large to solve in a reasonable time, but a 3-day look-ahead does provide substantial improvement over the base case for the RTS-GMLC; see Guerra et al.^31^); all other simulations used a 1-day look-ahead horizon.

Several factors have been suggested for siting storage in power systems (e.g., proximity to load or VRE,^27^ congestion/transmission sensitivity^48^). We considered five different factors by adjusting the 5-bus system configuration and analyzing the impacts of these factors on optimal siting locations for LDES. The factors included VRE location, VRE aggregation, load location, SDES location, and transmission capacity. To determine optimal LDES siting for a given configuration, we ran six simulations: one without LDES and five with the LDES sited at a different bus each iteration.

For the first four factors, we created five different system configurations for each factor by changing the placement of system components. These included the following tests.

(1) VRE location: Moving the wind generator that was originally on Bus 5 to a different bus (Figure 2A). If the bus that the VRE generator was placed on already had a VRE generator (Buses 2 and 3), that bus’s original generator was moved to Bus 5 to ensure that the VRE was dispersed throughout the system. This wind generator was chosen because Bus 5 had the least transfer capacity of transmission lines, so moving this generator could shift transmission requirements throughout the grid.
(2) VRE location and aggregation: Placing all three VRE generators in the original 5-bus system on a single bus (Figure 2B).
(3) Load location: Moving the load that was originally on Bus 4 (and the largest load in the system) to a different bus (Figure 2C). If the bus the load was placed on already had a load (Buses 2 and 3), that bus’s original load was moved to Bus 4 to ensure the load was still dispersed throughout the system.
(4) SDES location: Moving the SDES that was originally on Bus 1 to a different bus (Figure 2D).

In addition, to validate these results on a larger system, we also ran nine simulations in the RTS-GMLC. The first included no LDES in the system and the other eight simulations involved placing LDES (4,000 MW power capacity, 13.5 h of duration) at the bus with i) the largest load; ii) the largest net load; iii) the largest amount of VRE; iv) the largest average LMP (based on the results of the simulation without LDES; also corresponded to the largest standard deviation LMP); v) the largest closeness centrality returned by Graphs.jl^49^ (a topological metric that considers how “close” a bus is to the other buses in the system which we weight by transmission capacity), and vi - viii) an arbitrary bus containing high VRE, higher transfer capacity, and fewer number of congested hours seen at the bus. Look-ahead horizons were 1-day and 3-day for the case without LDES and the cases with LDES, respectively. See Table S2.

#### Additional notes

In our simulations presented in Figure 2, we note that the run with LDES on Bus 3 with the aggregated VRE on bus 4 initially resulted in an infeasible solution on one of the simulated days partway through the year. We resolved this problem by adding slack variables to the transmission constraints, where the slack variables are heavily penalized in the objective, and this resolved the infeasibility. This does mean that the production cost value for that simulation may be a slight under estimate.

For the 5-bus system simulations in the manuscript, we used the wind-driven system. However, the RTS system was run with the PV-driven case to address both types of energy mix, e.g., wind- and solar PV-driven. To verify if the results from the wind-driven case could potentially be applied to the PV-driven case of the RTS system, we also ran a set of simulations for the 5-bus system PV-driven case to see if the results were similar between the wind-driven and PV-driven cases. The PV-driven case also has a wind generator located on Bus 5 (the same as the wind-driven case) so we ran a similar set of simulations as we did with the wind-driven case where we moved the wind generator from bus 5 to the other buses and then ran simulations for LDES being located at each Bus but for the PV-driven case. These results are included in Table S1 and were overall similar to the results of the wind-driven case (but with higher overall production cost—likely a result of the PV-driven case having overall less VRE capacity.).

The RTS-GMLC simulations included a metric called “closeness centrality”. Closeness centrality is a topological metric equal to the inverse of the sum of the length of the shortest paths from a node to every other node^50^^,^^51^ (see also the Graphs.jl documentation for the function “closeness_centrality”). In this case, we weight distances by the inverse of the interconnection capacity (i.e., two buses connected by a line with a 2000 MW rating would be considered “closer” than two buses connected by a line with a 1000 MW rating). Because this measure considers the inverse of the sum of the distances, the smallest sum of shortest path lengths to each other node will have the largest closeness centrality. Thus, Basov could be considered the most central node in the graph because it has the highest closeness centrality value (as returned from Graphs.jl^49^; note that this is a normalized value).

For the RTS-GMLC results shown in Figure 4, the “normalized congested hours” is an estimate for the number of hours during the year that a bus is “congested”. This value is equal to the sum over all lines and tap transformers connected to a bus of the number of hours each line or tap transformer is at 100% of its rating over the course of a year, divided by the total number of lines and tap transformers connected to the bus. For instance, if a bus has three lines and zero tap transformers connected to it, and one line is at 100% of the rating for 20 h of the year, another line for 10 h of the year, and the last line for 0 h of the year, then the value reported in this column would be (20 + 10 + 0)/3 = 10. This normalized value is used because different buses had different numbers of connected lines. Note that Tap transformers are treated as components in PowerSimulations.jl. In the PCMs of PowerSimulations.jl in Sienna, tap transformers use the same optimization model as lines, and they are, like lines, contained on an edge or arc of the network. Under the network model (i.e., the DC approximation) we used for transmission, tap transformers operate as interconnection capacity within the PCM and can transfer flow in and out of a bus. While tap transformers are physically different from lines and have a different role in the grid, for the purposes of the PCM model, they are treated similarly to lines, and it is important to incorporate their line rating in the interconnection capacities.

### Quantification and statistical analysis

Means and standard deviations for LMPs are reported in Figure 2 and in Tables S1 and S2. These are based on all simulation time points (*n* = 8400) for their respective buses.

## Supplemental information

Document S1. Figure S1

Table S1. 5-bus system results Legend: Production costs and average LMPs for different simulations of the 5-bus system. Several simulations were performed to analyze the impact of system configuration on LDES siting. The “Test” column reports what was changed and how/where that change occurred. “LDES Location” indicates where the LDES component was sited for the simulation. “PC (Million USD)” is the total production cost for the 350 simulated days in million US Dollars. “Avg LMP (USD/MWh)” is the average LMP in USD per MWh that is computed from running a simulation for the same system configuration with no LDES components and computing the LMP from those simulation results. Tests included (1) moving the wind generator from Bus 5 to other buses in the system (if another bus already had a VRE generator; that generator was moved to Bus 5); (2) aggregating all three VRE generators onto one node; (3) moving the largest load from Bus 4 to a different bus (if a bus already had a load, that load was also moved to Bus 4; (4) moving the SDES component to a different bus in the system; (5) restricting all transmission line capacities by a set factor; and (6) moving the wind generator from Bus 5 to the other buses, but with the PV-driven case instead of the wind-driven case.

Table S2. RTS-GMLC Metrics Legend: Table containing different metrics for each bus in the RTS system. Each column is colored by a color gradient with red being the lower values in the column and blue the higher values. Column names correspond to: (1) “VRE” – the amount of VRE available at the bus over the simulation horizon in GWh; (2) “VRE_int” – the amount of VRE used at the bus in the simulation in GWh. “VRE” minus “VRE_int” is the amount of energy curtailed at that bus; (3) “load” – the total load at the bus over the simulation horizon in GWh; (4) “netload” – the netload at the bus over the simulation horizon in GWh (equivalent to “load” minus “VRE”); (5) “int_cap” – the interconnection capacity at the bus in MW; equivalent to the sum of the capacity of all lines and tap transformers connected directly to the bus; (6) “100lines” – an estimate for the number of hours during the year that a bus is “congested” (see STAR Methods; this is the normalized congested hours); (7) “LMP_avg” – the average LMP in SD / MWh based on the simulation with no LDES; (8) “LMP_std” – the standard deviation of the LMPs in USD/ MWh based on the simulation with no LDES; (9) “avg_int_cap” – the average capacity factor of transmission connected to a bus. This capacity factor of transmission for a bus at a given hour is computed by summing the absolute value of transmission usage for all lines and tap transformers connected to a bus and dividing by sum of the total capacity of all lines and tap transformers connected to the bus; (10) “closeness_centrality” – a topological metric for how “connected” each node is to all the other nodes in the system (see section 5.1). This value was computed using Graphs.jl in Julia [S3].
