# Supplementary material for: Toward understanding the complexity of long-duration energy storage siting in high renewable power grids
Source: iScience. 2025 May 2;28(6):112571. doi: 10.1016/j.isci.2025.112571 (PMC12145847; doi:10.1016/j.isci.2025.112571)
Supplement: Document S1. Figure S1 [file mmc1.pdf]

iScience, Volume *28*

Supplemental information

Toward understanding the complexity of long-duration energy storage siting in high renewable power grids

David L. Cole, Sourabh Dalvi, Victor M. Zavala, and Omar J. Guerra

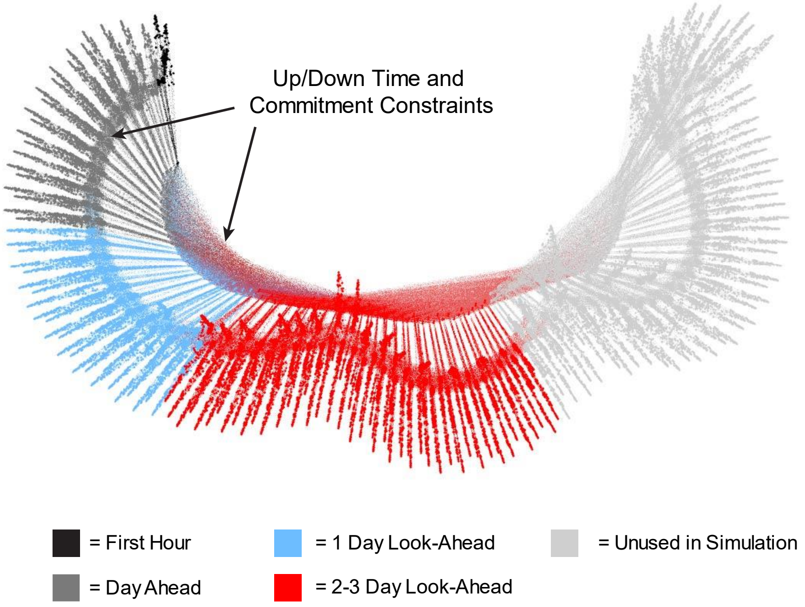

Figure S1 – [Visualization of the graph structure of the RTS system] – Related to Figure 1. Each bus at each time point is represented by a node, and each transmission line or tap transformer at each time point is represented by a node. Variables corresponding to that object are then stored on that node. Edges are formed in the graph by adding the constraints in the PCM. For example, for a bus containing a thermal generator, the node representing that bus at time t will be linked to the node representing that bus at time t-1 and the node representing that bus at time t+1 because of the commitment constraints of that thermal generator. Graphs are created based on the Julia package Plasmo.jl [S1] using the NFAPowerModel (pipe and bubble) [S2]

References

[S1] Jalving, J., Shin, S. and Zavala, V.M. A graph-based modeling abstraction for optimization: concepts and implementation in Plasmo.jl. *Math. Prog. Comp.* 14, 699-747.

[S2] Lara, J.D., Barrows, C., Thom, D., Dalvi, S., Callaway, D.S. and Krishnamurthy, D. 2024. PowerSimulations.jl – a power systems operation simulation library. *arXiv:*2404.03074.

[S3] Fairbanks, J., Besançon, M., Schölly, S., Hoffiman, J., Eubank, N. and Karpinski, S. JuliaGraphs/Graphs.jl: an optimized graphs package for the Julia programming language. (2021). https://github.com/JuliaGraphs/Graphs.jl/ (Accessed 5/30/24).
